# Identification of CD8+ T cell infiltration-related genes and their prognostic values in cervical cancer

**DOI:** 10.3389/fonc.2022.1031643

**Published:** 2022-10-31

**Authors:** Xiaopeng Shen, Chunguang Wang, Meng Li, Sufen Wang, Yun Zhao, Zhongxian Liu, Guoping Zhu

**Affiliations:** ^1^ College of Life Sciences, Anhui Normal University, Wuhu, Anhui, China; ^2^ Department of Pathology, The First Affiliated Hospital of Wannan Medical College, Wuhu, Anhui, China

**Keywords:** CD8+ T cells, cervical cancer, prognosis, IGSF6, TLR10, FCRL3, IFI30

## Abstract

Cervical cancer is a female-specific cancer with relatively high morbidity and mortality. As known to all, immune cell infiltrations in the cancer microenvironment are closely related to the cancer diagnosis and prognosis. Here we revealed that the CD8+ T cell infiltration was significantly upregulated in cervical cancer versus normal cervix uteri samples. Through univariate and multivariate cox analyses, we discovered that the CD8+ T cell infiltration was the only independent beneficial factor for the prognosis of cervical cancer. To explore the genes associated with the CD8+ T cell infiltration in cervical cancer, we performed the WGCNA analysis on the differentially expressed genes (DEGs) of cervical cancer versus normal cervix uteri tissues. As a result, 231 DEGs were found to be associated with CD8+ T cell infiltration in cervical cancer. Subsequently, with the Cytoscape analysis, we identified 105 hub genes out of the 231 DEGs. To further explore the genes that might be responsible for the prognosis of cervical cancer, we performed a univariate cox analysis followed by a LASSO assay on the 105 hub genes and located four genes (IGSF6, TLR10, FCRL3, and IFI30) finally. The four genes could be applied to the prediction of the prognosis of cervical cancer, and relatively higher expression of these four genes predicted a better prognosis. These findings contributed to our understanding of the prognostic values of CD8+ T cell infiltration and its associated genes in cervical cancer and thus might benefit future immune-related therapies.

## Introduction

Cervical cancer, which primarily arises from the cervix, is the fourth most common and death-causing cancer in women worldwide (1). According to recent Global Cancer statistics, there were about 604,000 new cases and 342,000 deaths of cervical cancer in 2020 (2). The incidence and mortality of cervical cancer were both significantly higher in developing countries, which were 7~10 and 18 times exceeding these in developed countries, respectively (2). Human papillomavirus (HPV) is believed to be the primary cause of cervical cancer. Over 90% of squamous cervical cancer were diagnosed to be HPV positive (3). Thus, the application of the HPV vaccine largely constrained the incidence of cervical cancer in developed countries, but not in developing countries due to the limited vaccination rate (4, 5). Conventional therapy approaches against cervical cancer include surgery, radiation, chemotherapy, targeted drug therapy, and immunotherapy (6–9). Besides these, many efforts have been made to apply Chimeric Antigen Receptor T-Cell (CAR-T) immunotherapy to the therapy against cervical cancer (10–12). The overall therapy outcomes are closely associated with therapy strategies designed based on the specific gene profiles of each individual.

Immune cell infiltration in cancer is an indispensable microenvironmental factor that has been widely applied to prognosis prediction and immunotherapy (13–15). The infiltrations of activated B cells, memory effector T cells, eosinophils, and plasmacytoid dendritic cells were shown to be associated with a better prognosis in cervical cancer (16). The composition of immune cell infiltration might even allocate cervical cancer patients into different subtypes that displayed distinct prognostic outcomes, thus implying the importance of immune cell infiltration (17). Moreover, some therapy strategies, for example, neoadjuvant chemotherapy, work at least partially by reprogramming the immune microenvironment of cervical cancer (17). Some genes have been reported to manipulate the immune cell infiltrations in certain cancers and thus serve as prognostic biomarkers, such as TREM2, HSF1, and GIMAP4 (18–20). Nevertheless, the role of activated CD8+ T cells in the prognosis of cervical cancer and the genes that are associated with CD8+ T cells remains uncertain.

In this study, we evaluated the changes in immune cell infiltration of cervical cancer versus normal tissues. We found that the CD8+ T cell infiltration was the only independent beneficial factor for the prognosis of cervical cancer. Four genes (IGSF6, TLR10, FCRL3, and IFI30) were found to be CD8+ T cell infiltration-related genes. The four genes could be applied to the prediction of cervical cancer prognosis.

## Materials and methods

### General information of datasets

The clinical information and RNA sequencing (RNA-seq) data of the Cervical Squamous Cell Carcinoma and Endocervical Adenocarcinoma (CESC) were downloaded from The Cancer Genome Atlas (TCGA), which included 306 cervical cancer tissues and 3 normal control samples (termed TCGA-CESC cohort). The clinical information of the TCGA-CESC cohort was shown in **Table 1**. The data of 10 normal cervical tissue samples were downloaded from the Genotype Tissue Expression (GTEx) database. When analyzing the data from TCGA and GTEx together, we directly downloaded a merged expression matrix of these two datasets from the UCSC Xena database instead. The GSE151666 dataset in the Gene Expression Omnibus (GEO) database, which included the RNA-seq data of 68 pre-treatment primary cervical cancer samples, was used as a validation cohort. The expression matrixes retrieved from the TCGA and GTEx databases were in count format, while the expression matrix from the GSE151666 dataset was in FPKM format.

**Table 1:** Clinical characteristics of the TCGA-CESC cohort.

Characteristics	Patients (*N* = 306)	
	*N*	%
**Gender**		
Female	306	100%
**Stage**		
I	162	52.90%
II	69	22.50%
III	46	15.00%
IV	22	7.20%
NA	7	2.30%
**T**		
T1	140	45.80%
T2	72	23.50%
T3	21	6.90%
T4	10	3.30%
TIS	1	0.30%
TX	17	5.60%
NA	45	14.70%
**N**		
N0	134	43.80%
N1	61	19.90%
NX	66	21.60%
NA	45	14.70%
**M**		
M0	116	37.90%
M1	11	3.60%
MX	129	42.20%
NA	50	16.30%
**Race**		
American Indian or Alaska Native	7	2.30%
Asian	20	6.50%
Black or African American	31	10.10%
Native Hawaiian or other pacific islander	2	0.70%
white	210	68.60%
NA	36	11.80%
**Age**		
<=50	188	61.40%
> 50	118	38.60%
**Vital status**		
ALIVE	233	76.10%
DEAD	73	23.90%
**Survival time**		
Long(>5yeas)	47	15.40%
Short(<5years)	259	84.60%

TCGA, The Cancer Genome Atlas; CESC, cervical squamous cell carcinoma and endocervical adenocarcinoma; NA, not applicable.

### RNA-seq data processing and differentially expressed gene determination

Differentially expressed genes (DEGs) were determined by the “DESeq” software. The adjusted P value <= 0.01 and |log2(FoldChange)| > 2 were used as the cutoff criteria. The gene ontology (GO) analysis was performed using the “clusterProfile” package in R (version 4.1.0) and the Kyoto Encyclopedia of Genes and Genomes (KEGG) analysis was performed at The Database for Annotation, Visualization and Integrated Discovery (DAVID) platform (https://david.ncifcrf.gov/). P value < 0.05 was used as the significance threshold. GO results were plotted with the “GOplot” package in R, while KEGG results were plotted using the “ggplot2” package in R.

### CIBERSORT analysis

The CIBERSORT analysis was performed to determine immune cell infiltrations as described previously (21). The results of the CIBERSORT analysis included the relative infiltration levels of B cells (naïve B cells, memory B cells, and plasma cells), T cells (CD8+ T cells, naïve CD4 T cells, resting memory CD4+ T cells, activated memory CD4+ T cells, follicular helper T cells, regulatory T cells, and gamma delta T cells), NK cells (resting NK cells and activated NK cells), monocytes, macrophages (M0 macrophages, M1 macrophages, and M2 macrophages), dendritic cells (resting dendritic cells and activated dendritic cells), mast cells (resting mast cells and activated mast cells), eosinophils, neutrophils. P value < 0.05 was used as the significance threshold.

### Weighted gene co-expression network analysis (WGCNA)

The DEGs between normal and cervical cancer groups in the TCGA-CESC cohort were subjected to WGCNA, which was performed using the “WGCNA” package in R. We used 0.9 as the cutoff for the scale-free fit index and obtained soft-thresholding power = 2 with the pickSoftThreshold function in the “WGCNA” package. By setting the soft-thresholding power = 2, the DEGs were allocated into 15 modules, which were visualized in a cluster dendrogram. The correlation values and significances between the modules and immune cell infiltrations were calculated and displayed in heatmaps.

### Protein-protein interaction analysis

The protein-protein interaction was analyzed in the STRING database (https://cn.string-db.org/) and plotted with the Cytoscape software. The hub genes in the protein-protein interaction network were determined by 12 algorithms in the cytoHubba of Cytoscape, respectively, which were Betweenness, BottleNeck, Closeness, ClusteringCoefficient, Degree, DMNC, EcCentricity, EPC, MCC, MNC, Radiality, and stress. The shared hub genes by all 12 algorithms were considered to be the candidate hub genes, which were determined and plotted with the “UpSetR” package in R.

### Univariant and multivariant Cox analysis

To evaluate the clinical significance of cervical cancer infiltrated immune cells, univariant and multivariant Cox analyses were performed with the “coxph” function in the “survival” package. As to the clinical significance of the hub genes determined by the WGCNA and Cytoscape, univariant Cox analysis was performed with the “survival” package in R. P value < 0.05 was used as the cutoff to select significant gene candidates, which were then subjected to the least absolute shrinkage and selection operator (LASSO) analysis. The LASSO analysis was performed using the “glmnet” package in R to further filter the genes that significantly affected the prognosis of cervical cancer.

### Receiver Operating Characteristic (ROC) analysis

The expressions of selected genes along with the overall survival data of the TCGA-CESC cohort were used to evaluate the predictive values of these genes in the prognosis of cervical cancer. The ROC curve and areas under the ROC curve (AUC) were generated by the “timeROC” package in R.

### Overall survival (OS) analysis

291 patients in the TCGA-CESC cohort, whose OS was larger than zero, were included in the OS analysis. The OS was examined by log-rank and Kaplan-Meier plots. When studying the impacts of target genes on the OS, the patients were equally divided into two groups (i.e. “high” versus “low”) based on their median expressions. The OS curves were plotted with the “survival” and “survminer” packages in R. P value < 0.05 was considered to be significant.

### Immunohistochemical staining

Tumor and para-carcinoma tissues of cervical cancer were embedded in paraffin and then sectioned. Immunohistochemical staining was performed using an Immunohistochemistry Kit (Sangon Biotech, Cat# D601037) according to the manufacturer’s instructions. In brief, slides were first dewaxed with xylene and rehydrated with gradient ethanols. Subsequently, the slides were subjected to antigen retrieval with citrate solution in boiling water. The slides were then blocked with bovine serum albumin (BSA) and incubated with primary antibodies overnight. After that, the slides were incubated with HRP-conjugated secondary antibodies and then reacted with the DAB solution in the kit. Finally, the slides were incubated with hematoxylin. The staining results were observed with a Leica DMi8 fluorescence microscope. The antibodies used in this study were as follows: IGSF6 mAb (Santa Cruz, Cat# sc-377053), TLR10 mAb (Santa Cruz, Cat# sc-293300), FCRL3 mAb (Santa Cruz, Cat# sc-365706), IFI30 mAb (Santa Cruz, Cat# sc-393507), and Goat Anti-Mouse HRP secondary antibody (biosharp, Cat# BL001A). The cervical cancer samples used in this study were obtained with written informed consent from patients. Our study was approved by the Ethics Committee of Anhui Normal University.

## Results

### Identification of immune cell infiltrations in cervical cancer

**Figure 1:**
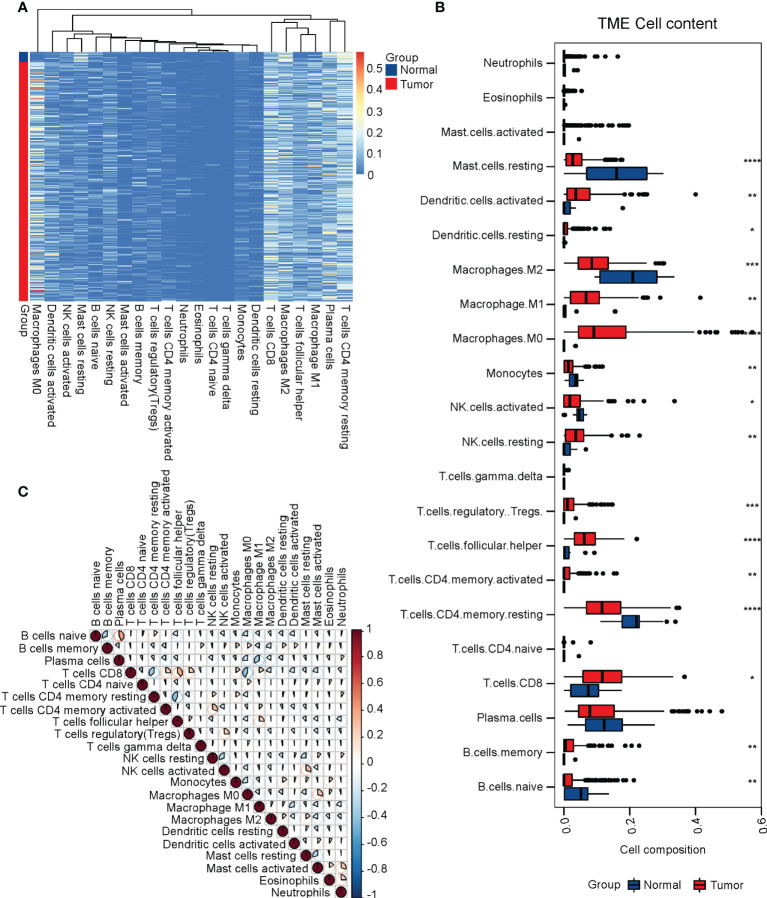
Identification of immune cell infiltrations in cervical cancer. To evaluate immune cell infiltrations, we performed a CIBERSORT analysis on the TCGA-CESC cohort along with normal cervix samples from the GTEx. **(A)** The heatmap of immune cell infiltrations in normal and cervical cancer groups. **(B)** The relative levels of immune cell infiltrations in normal and cervical cancer groups. **(C)** The correlation of immune cell infiltrations in normal and cervical cancer groups. *P < 0.05; **P < 0.01; ***P < 0.001; ****P < 0.0001.

We downloaded the RNA-seq data of the TCGA-CESC cohort along with normal cervix uteri samples from the GTEx database. To investigate the immune cell infiltrations in normal cervix uteri and cervical cancer, we performed a CIBERSORT analysis on the RNA-seq data. Among the 22 infiltrated immune cell types, activated dendritic cells, resting dendritic cells, M0 and M1 macrophages, resting NK cells, regulatory T cells, follicular helper T cells, activated memory CD4+ T cells, CD8+ T cells, and memory B cells were significantly upregulated, while resting mast cells, M2 macrophages, monocytes, activated NK cells, resting memory CD4+ T cells, and naïve B cells were significantly downregulated. (**Figures 1A, B**) The infiltrated immune cells were differentially correlated with each other. (**Figure 1C**) Among these, naïve B cells were positively related to plasma cells but negatively related to memory B cells, implying the diverse B cell trends in cervical cancer. CD8+ T cells were negatively correlated with naïve CD4+ T cells and M0 macrophages but positively correlated with follicular helper T cells.

### CD8+ T cell infiltration was associated with the prognosis of cervical cancer

**Figure 2:**
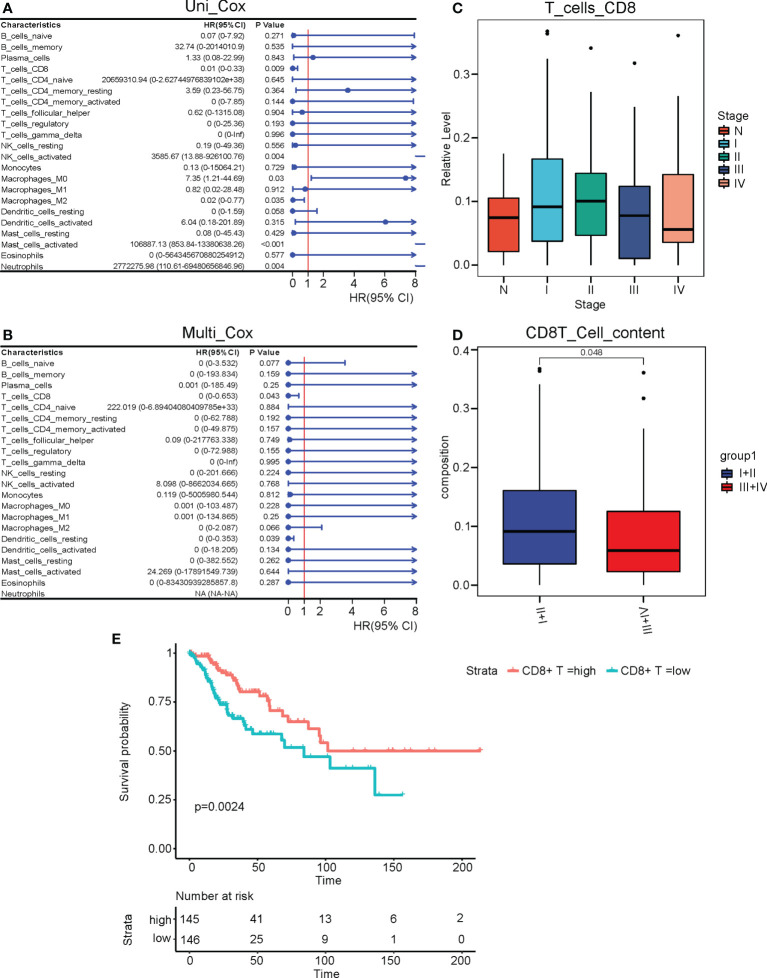
CD8+ T cell infiltration was associated with the prognosis of cervical cancer. **(A)** Univariant and **(B)** multivariant Cox regression analyses performed on the infiltrated immune cells in the TCGA CESC cohort. **(C)** The relative levels of CD8+ T cell infiltrations at different clinical stages of cervical cancer. **(D)** The relative levels of CD8+ T cell infiltrations at stages III and IV versus stages I and II. **(E)** The survival curve of cervical cancer patients with high and low levels of CD8+ T cell infiltrations. N, Normal; I, stage I; II, stage II; III, stage III; IV, stage IV; I+II, stage I and II; III+IV, stage III and IV.

To determine which immune infiltrations were associated with the prognosis of cervical cancer, we performed both univariant and multivariant Cox regression analyses on the TCGA-CESC cohort. The result of univariant cox analysis suggested that the infiltration of activated NK cells, M0 macrophages, activated mast cells, and neutrophils were hazard factors for the overall survival (OS) of cervical cancer patients, while the CD8+ T cells and M2 macrophages were beneficial factors for the OS. (**Figure 2A**) Moreover, multivariant cox analysis suggested that the CD8+ T cells and resting dendritic cells were independent beneficial factors affecting the OS of cervical cancer. (**Figure 2B**) It was worth noting that the infiltration of resting dendritic cells was not a significant factor in the univariant cox analysis (**Figure 2A**), though it was significant in the multivariant cox analysis (**Figure 2B**). Thus, we focused on the CD8+ T cell infiltration in subsequent studies. We next plotted the relative levels of the CD8+ T cell infiltration in normal cervix uteri and cervical cancer at different histological stages. The CD8+ T cell infiltration was gradually elevated at stages I and II compared to normal ones but dropped at stages III and IV compared to stage II. (**Figure 2C**) The mean level of CD8+ T cell infiltration at stages III and IV was significantly lower than that of stages I and II. (**Figure 2D**) By dividing the TCGA-CESC cohort into two groups based on the level of CD8+ T cell infiltration, we found that the patients with a higher CD8+ T cell infiltration showed a remarkable improvement in their OS (P=0.0024). (**Figure 2E**) Therefore, the CD8+ T cell infiltration, which was first elevated at the initial stages of cervical cancer and then dropped at later stages, was an independent beneficial factor for the OS of cervical cancer.

### The altered DEGs, biological processes, and pathways in cervical cancer

**Figure 3:**
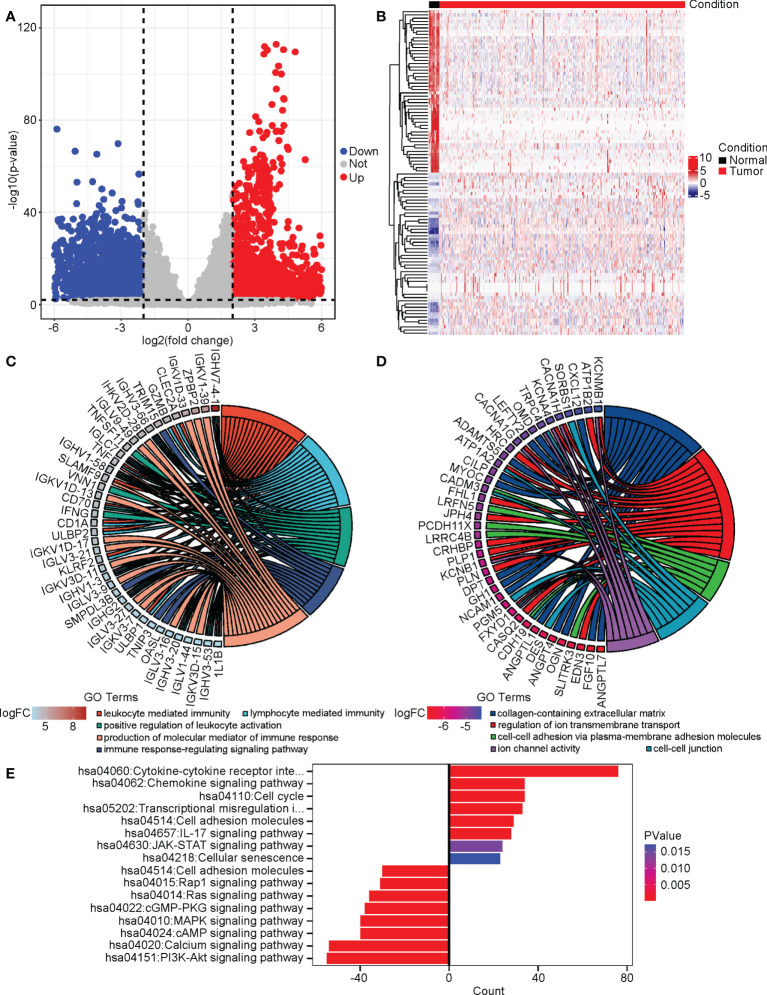
The altered DEGs, biological processes, and pathways in cervical cancer. We analyzed the DEGs between cervical cancer and normal tissues and then performed GO and KEGG analyses on these DEGs. **(A)** The volcano plot of DEGs. **(B)** The heatmap of the top 50 upregulated and downregulated DEGs. **(C)** The upregulated GO terms and their associated DEGs. **(D)** The downregulated GO terms and their associated DEGs. **(E)** The altered KEGG pathways.

We next studied the differentially expressed genes (DEGs) in cervical cancer compared to normal tissues. We performed a DEG analysis using the “DESeq2” package in R on the merged RNA-seq data of the cervix uteri in the GTEx and the TCGA-CESC cohort. |log2(FoldChange)| > 2 and adjusted P value < 0.01 was used as the cutoff. As a result, we identified 2111 upregulated and 2160 downregulated DEGs. (**Figure 3A**) We plotted a heatmap on the top altered 50 upregulated and 50 downregulated DEGs. (**Figure 3B**) Next, we performed gene ontology (GO) analyses on the upregulated and downregulated DEGs, respectively. The GO terms enriched with the upregulated DEGs included leukocyte-mediated immunity, lymphocyte-mediated immunity, positive regulation of leukocyte activation, immune response-regulating signaling pathway, and production of molecular mediator of the immune response. (**Figure 3C**) As to the downregulated DEGs, the GO terms included collagen-containing extracellular matrix, regulation of ion transmembrane transport, cell-cell adhesion *via* plasma-membrane adhesion molecules, cell-cell junction, and ion channel activity. (**Figure 3D**) KEGG analysis suggested that the cytokine/chemokine-involved pathways, JAK-STAT signaling, and cell cycle were promoted, while cell adhesion, PI3K-AKT, calcium, cAMP, etc. related pathways were repressed. (**Figure 3E**)

### Identification of four genes that were involved in the CD8+ T cell infiltration in cervical cancer

**Figure 4:**
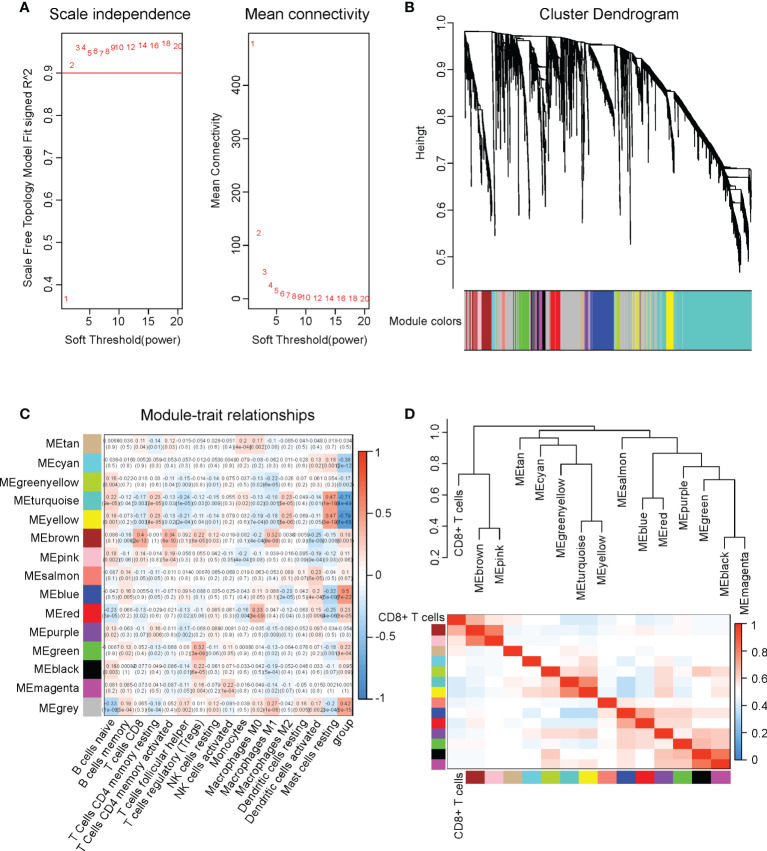
Identification of four genes that were involved in CD8+ T cell infiltration in cervical cancer. We performed WGCNA on the DEGs in cervical cancer versus normal tissues. **(A)** Soft-thresholding power decision using 0.9 as the cutoff for the scale-free fit index in WGCNA. **(B)** The cluster dendrogram of WGCNA. **(C)** The correlations between all immune infiltrations and different modules in WGCNA. **(D)** The hierarch clustering on the CD8+ T cell infiltration and all WGCNA modules.

Subsequently, we determined the CD8+ T cell infiltration-associated DEGs using the weighted gene co-expression network analysis (WGCNA). We used 0.9 as the cutoff for the scale-free fit index and chose soft-thresholding power = 2 for the WGCNA. (**Figure 4A**) All DEGs were classified into 15 modules and assigned different colors. The DEGs in grey color were the genes excluded from all the modules. (**Figure 4B**) We performed correlation assays on the modules and immune cell infiltrations in cervical cancer. Among all modules, module brown (MEbrown) exerted the highest correlation value (0.4) with the CD8+ T cell infiltration (P=2E-13). (**Figure 4C**) When we performed the hierarch clustering on the CD8+ T cell infiltration and all WGCNA modules, we found that the CD8+ T cell stayed the closest to the MEbrown module. (**Figure 4D**) These results implied that the 231 DEGs in the MEbrown might be associated with the CD8+ T cell infiltration in cervical cancer.

**Figure 5:**
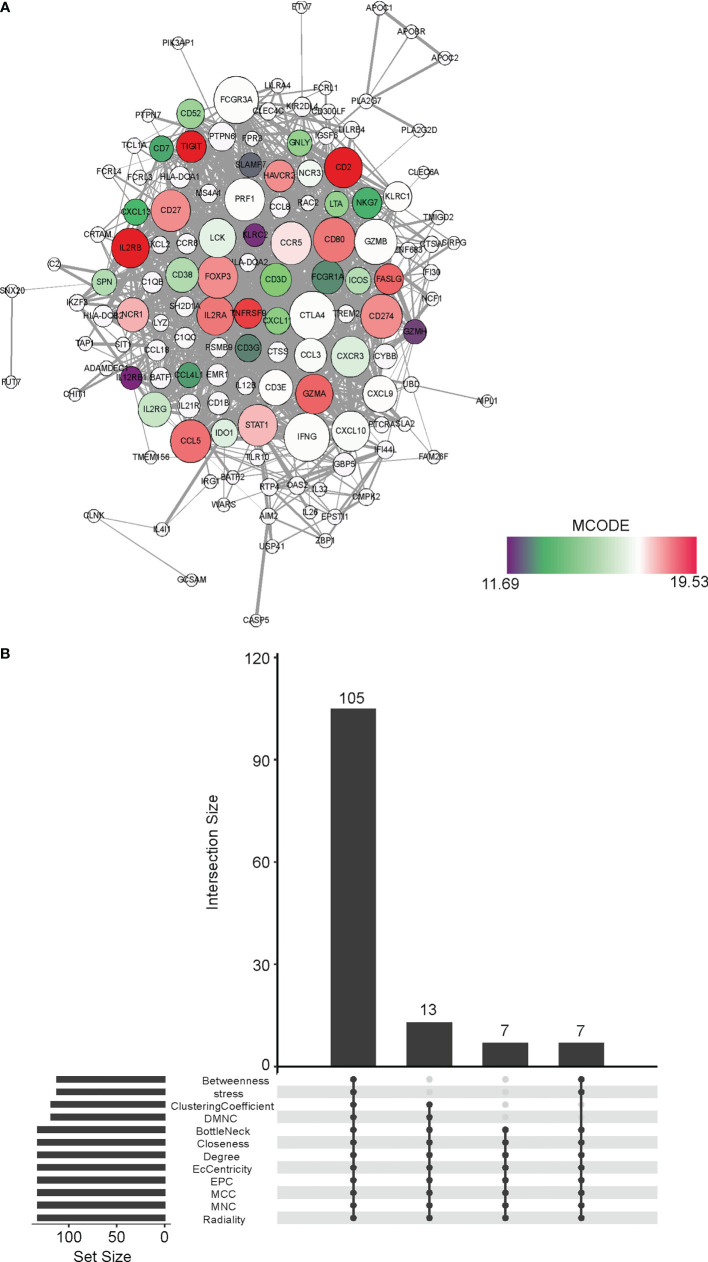
Identification of hub genes in the CD8+ T cell infiltration-related MEbrown module **(A)** The Protein-Protein Interaction of the DEGs belonging to the MEbrown module in WGCNA. The network was generated by the Cytoscape software. **(B)** The shared hubs genes determined by 12 different algorithms, which were Betweenness, stress, ClusteringCoefficient, DMNC, BottleNeck, Closeness, Degree, EcCentricity, EPC, MCC, MNC, and Radiality.

Next, we performed a Protein-Protein Interaction network assay on the 231 DEGs in the WGCNA MEbrown module. (**Figure 5A**) We determined hub genes using 12 different algorithms, which were Betweenness, stress, ClusteringCoefficient, DMNC, BottleNeck, Closeness, Degree, EcCentricity, EPC, MCC, MNC, and Radiality. Collectively, there were 105 hub genes shared by all the 12 algorithms, which were focused on in subsequent studies. (**Figure 5B**)

**Figure 6:**
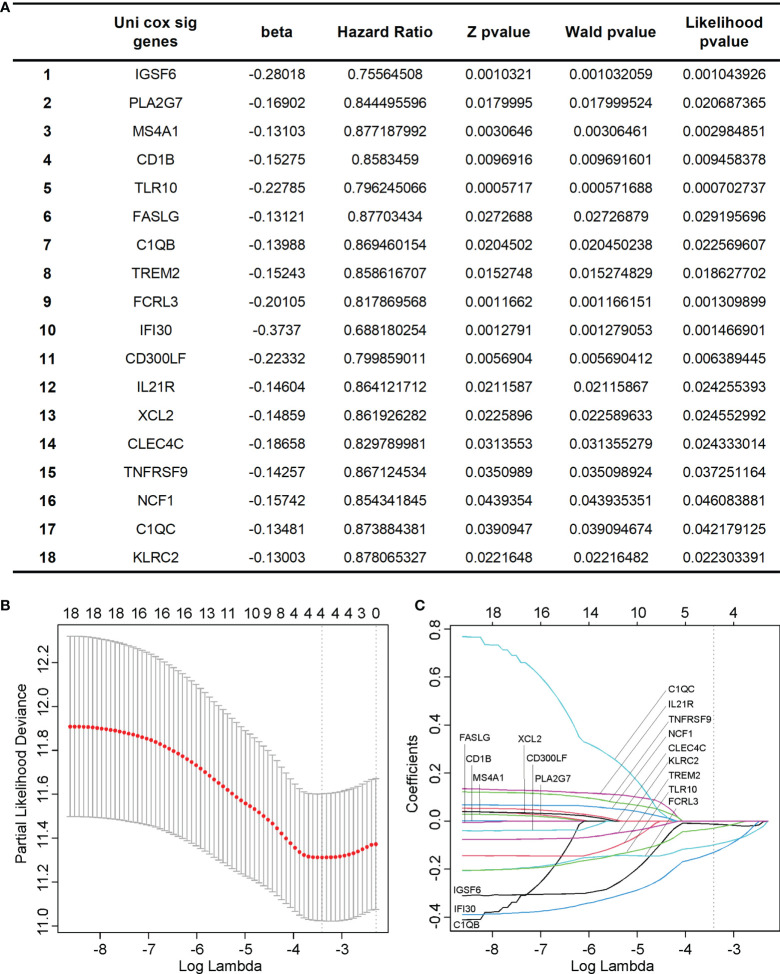
Identification of key hub genes significantly associated with the prognosis of cervical cancer. **(A)** We identified 18 hub genes that significantly affected the cervical cancer prognosis using univariant Cox analysis. **(B, C)** we performed LASSO analysis on the 18 candidate genes and finally got 4 genes, which were IGSF6, TLR10, FCRL3, and IFI30.

We performed a univariant Cox analysis to test the association between the 105 hub genes and the OS of cervical cancer. As a result, 18 genes significantly affected the cervical cancer prognosis. (**Figure 6A**) To further refine the genes, we performed LASSO analysis on these 18 candidate genes and finally got 4 genes, which were IGSF6, TLR10, FCRL3, and IFI30. (**Figures 6B, C**)

### The four CD8+ T cell-related genes were upregulated in cervical cancer and associated with prognosis

**Figure 7:**
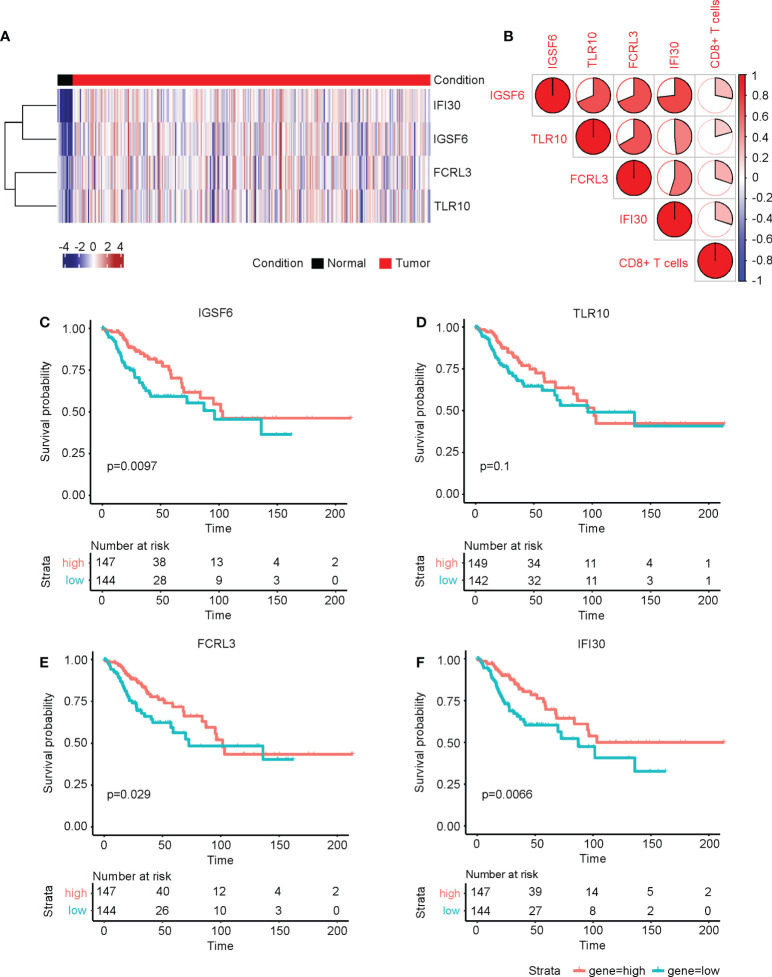
The four CD8+ T cell-related genes were upregulated in cervical cancer and associated with prognosis. **(A)** The heatmap of the relative levels of IGSF6, TLR10, FCRL3, and IFI30 in normal and cervical cancer tissues. **(B)** The correlations of IGSF6, TLR10, FCRL3, IFI30, and CD8+ T cell infiltration. The overall survival curves of **(C)** IGSF6, **(D)** TLR10, **(E)** FCRL3, and **(F)** IFI30 in cervical cancer.

**Figure 8:**
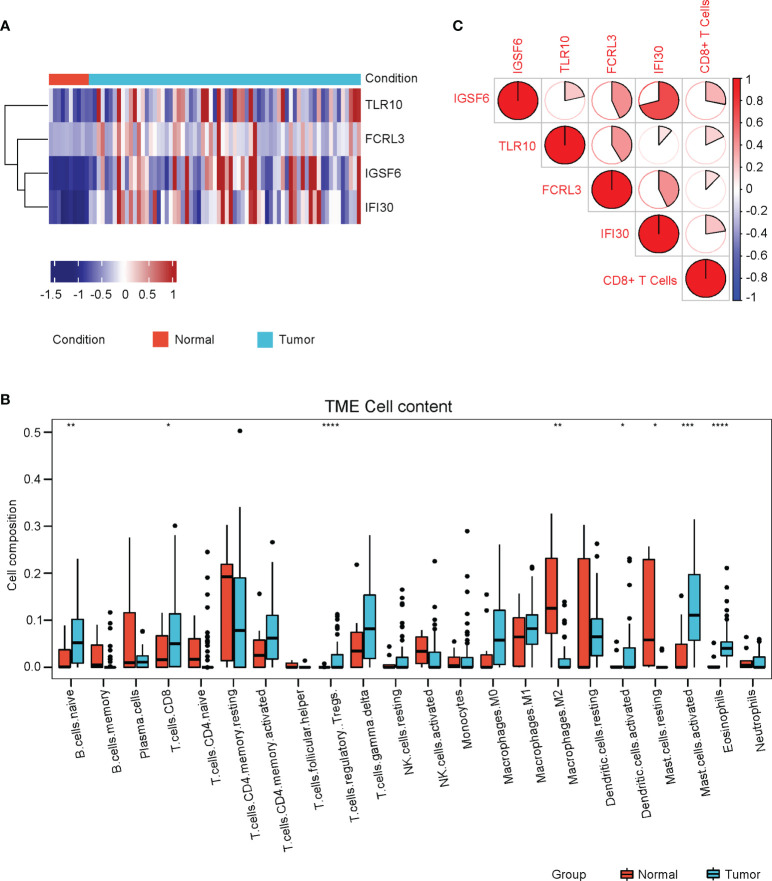
Validation of immune cell infiltrations and gene profile in the validation cohort. **(A)** The heatmap of the relative levels of IGSF6, TLR10, FCRL3, and IFI30 in normal and cervical cancer tissues in the validation cohort. **(B)** The relative levels of immune cell infiltrations in normal and cervical cancer groups in the validation cohort. **(C)** The correlations of IGSF6, TLR10, FCRL3, IFI30, and CD8+ T cell infiltration in the validation cohort. *P < 0.05; **P < 0.01; ***P < 0.001; ****P < 0.0001.

**Figure 9:**
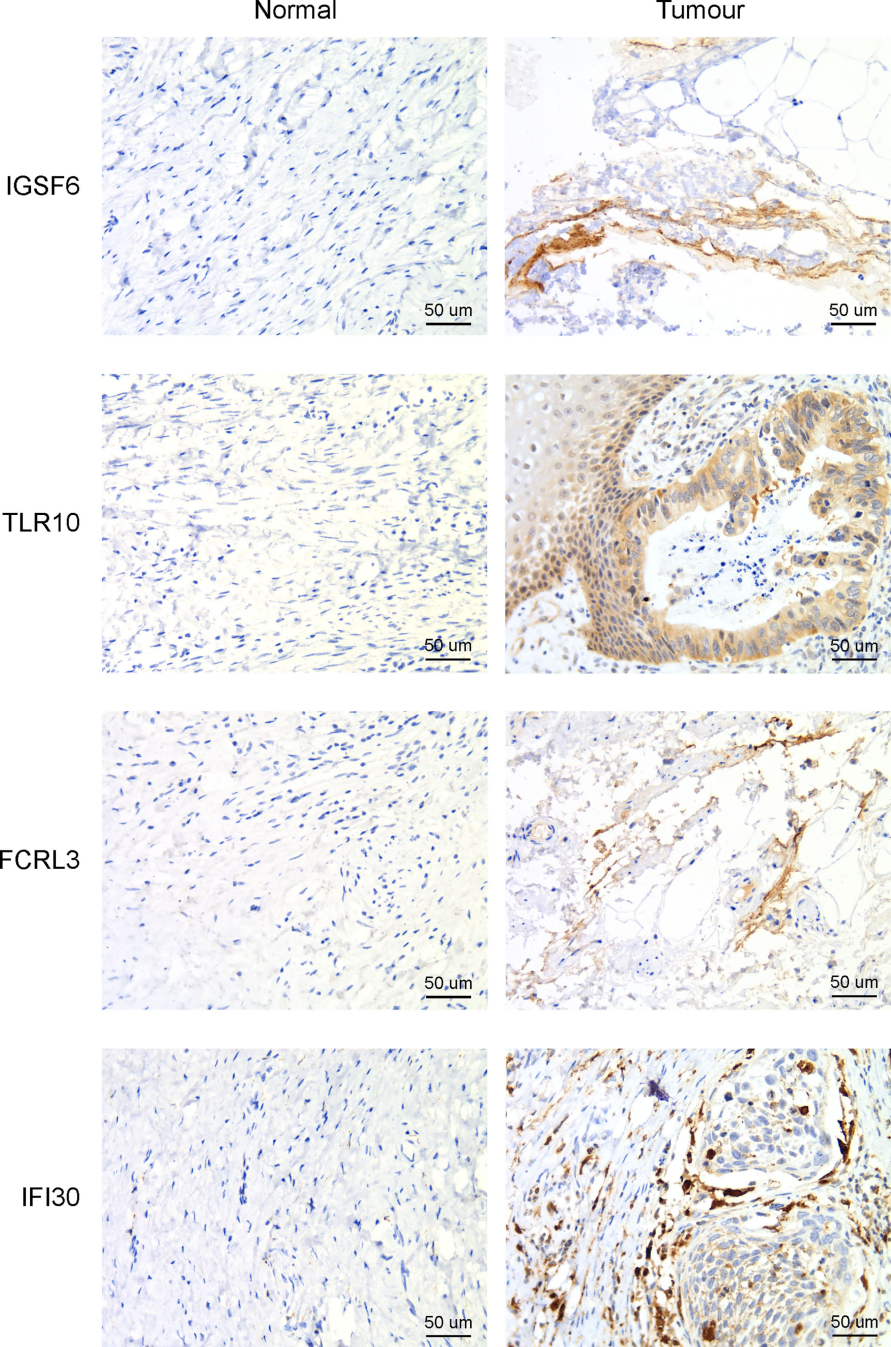
Immunohistochemistry to detect the four CD8+ T cell-related genes in cervical cancer and adjacent normal tissues.

The four genes derived from the LASSO test (**Figures 6B, C**) were all upregulated in cervical cancer. (**Figure 7A**) They were closely and positively correlated with the CD8+ T cell infiltration. (**Figure 7B**) Except for TLR10, high expressions of these genes all predicted a better OS. (**Figures 7C–F**) To validate the immune infiltration pattern, the expressions of IGSF6, TLR10, FCRL3, and IFI30, and the correlation between them, we downloaded the GSE151666 dataset from the GEO database as a validation cohort, which included 68 cervical cancer patients. GTEx normal cervix uteri samples were used as a control. Consistently, the expressions of IGSF6, TLR10, FCRL3, and IFI30 were upregulated in cervical cancer in the validation cohort. (**Figure 8A**) By CIBERSORT analysis, naïve B cells, CD8+ T cells, regulatory T cells, activated dendritic cells, activated mast cells, and eosinophils were significantly upregulated, while M2 macrophages and resting mast cells were downregulated. (**Figure 8B**) The four factors also showed close and positive correlations with the CD8+ T cell infiltration. (**Figure 8C**) To validate the expressions of IGSF6, TLR10, FCRL3, and IFI30 in cervical cancer, we performed immunohistochemistry against these genes, respectively. In line with the results from the transcriptomics, we identified stronger expressions of IGSF6, TLR10, FCRL3, and IFI30 in cervical cancer tissues compared to normal tissues. (**Figure 9**)

### The four CD8+ T cell-related genes could be a predictive factor for the prognosis of cervical cancer

**Figure 10:**
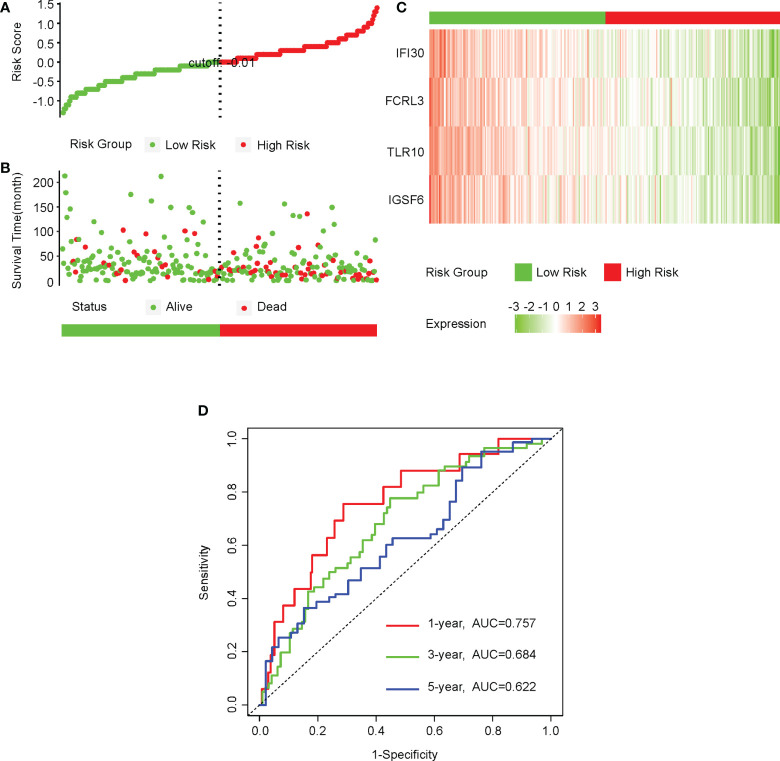
The four CD8+ T cell-related genes could be a predictive factor for the prognosis of cervical cancer. **(A)** We first performed a cox analysis on the CESC-TCGA cohort and equally divided the patients into low- and high-risk groups using the cutoff of -0.01 for the risk score. **(B)** The distributions of survival time of patients in the low- and high-risk groups. **(C)** The heatmap of the relative levels of IGSF6, TLR10, FCRL3, and IFI30 in the low- and high-risk groups. **(D)** The ROC curves showing the predictive value of the combinations of IGSF6, TLR10, FCRL3, and IFI30 in the prognosis of cervical cancer.

As the expressions of IGSF6, TLR10, FCRL3, and IFI30 were all upregulated in cervical cancer and the higher expressions of IGSF6, FCRL3, and IFI30 predicted better prognosis, we asked if the combination of these four genes was associated with the risks of cervical cancer. We first performed cox analysis on the TCGA-CESC cohort and equally divided the patients using the cutoff of -0.01 for the risk scores. (**Figure 10A**) Agreeing with this, the high-risk group included a relatively higher number of deaths, while the low-risk group displayed a relatively longer survival time. (**Figure 10B**) The expressions of all these four factors were remarkably lower in the high-risk group. (**Figure 10C**) To evaluate the predictive value in the prognosis of cervical cancer, we performed a ROC analysis with the expressions of these four factors in the TCGA-CESC cohort. The AUCs for 1, 3, 5, -year ROC curve were 0.757, 0.684, and 0.622, respectively. (**Figure 10D**)

## Discussion

Cervical cancer is among the top threats to the health and lives of females. Immune cell infiltrations in the cancer microenvironment have been proven to be an important factor in both diagnosis and prognosis prediction. In this study, we found that the CD8+ T cell infiltration was significantly upregulated in cervical cancer versus normal cervix uteri samples. Through univariate and multivariate cox analyses, we confirmed that the CD8+ T cell infiltration was an independent beneficial factor for the prognosis of cervical cancer. Next, we performed WGCNA on the DEGs of cervical cancer and identified the hub genes using the Cytoscape software, which might be associated with the CD8+ T cell infiltration. Subsequently, we performed univariate cox and LASSO analyses on the hub genes and got four genes (IGSF6, TLR10, FCRL3, and IFI30) finally. The four genes could be applied to the prediction of the prognosis of cervical cancer, and the higher expression of the four genes suggested lower risks for cervical cancer patients.

Immune cell infiltrations in cancer serve as a crucial tumor microenvironment factor that mediates tumor metabolism, immunotherapy outcome, and cancer prognosis prediction (13–15). Although the infiltration of activated B cells, memory effector T cells, eosinophils, and plasmacytoid dendritic cells have been reported to be associated with a better prognosis of cervical cancer (16), the effect of CD8+ T cell infiltration in cervical cancer was previously uncertain. We here discovered that the CD8+ T cell infiltration was significantly upregulated in cervical cancer compared to normal cervix uteri tissues in both the TCGA-CESC and GSE151666 cohorts. With univariate and multivariate cox analyses, we found that the CD8+ T cell infiltration was an independent beneficial factor for the prognosis of cervical cancer. These results suggested that the CD8+ T cell infiltration was evoked upon the occurrence of cervical cancer and served a protective role aimed at eliminating such tumor cells, which agreed with its role in cellular immunity. We determined the CD8+ T cell infiltration in cervical cancer at different histological stages. We found that the CD8+ T cell infiltration was truly upregulated at the early stages (I&II) of cervical cancer compared to normal tissues but significantly decreased at late stages (III&IV). It should be noted that although the CD8+ T cell infiltration level was elevated at early stages, these CD8+ T cells could not properly target tumor cells due to the immunosuppressive microenvironment within tumors. This also implied that the therapy against the immunosuppressive microenvironment at the early stages of cervical cancer might achieve better outcomes. As to the level decrease of the CD8+ T cell infiltration at late stages, a possible reason was that the overall immune system in the body was debilitated.

We also analyzed the DEGs in cervical cancer versus normal cervix uteri tissues. As a result, we identified 2111 upregulated and 2160 downregulated DEGs. We performed GO analyses on the upregulated and downregulated DEGs, respectively. In line with the immune cell infiltration analysis, the processes, such as leukocyte-mediated immunity, lymphocyte-mediated immunity, positive regulation of leukocyte activation, immune response-regulating signaling pathway, and production of molecular mediator of the immune response, were enriched in the upregulated DEGs. Likewise, the cytokine/chemokine-involved pathways were enriched in the KEGG pathway analysis of the upregulated DEGs. These results suggested that the upregulated DEGs in cervical cancer were involved in the regulation of immune cell infiltration, implying a necessity of revealing immune infiltration-related DEGs.

WGCNA is a powerful tool to locate candidate gene clusters (modules) that were related to external sample traits, for example, immune cell infiltrations here (22). We here adopted WGCNA to search for the genes that might be responsible for the CD8+ T cell infiltration changes in cervical cancer. As a result, we found that the 231 genes belonging to the brown module showed the highest correlation with the CD8+ T cell infiltration. To further filter the genes, we performed a protein-protein interaction analysis using the Cytoscape. By exploring hub genes using 12 different algorithms, we got 105 candidate hub genes. We next tested if these candidate hub genes were associated with the prognosis of cervical cancer. By univariant Cox and LASSO analyses, we finally got 4 genes (IGSF6, TLR10, FCRL3, and IFI30) that were closely correlated with both the CD8+ T cell infiltration and the prognosis of cervical cancer. The identification of diagnostic and prognostic biomarkers has brought significant advances to the clinical treatments of certain cancer types, for example, pancreatic ductal adenocarcinoma (PDAC). Currently discovered diagnostic biomarkers of PDAC include glycoproteins (23, 24), microRNAs (25), circulating tumor DNA, circulating tumor cells (26), and metabolomics (27). Some biomarkers have also been applied into guiding drug usage. For instance, the expression of human ENT1 was revealed as an important predictor for the responses to gemcitabine, a chemotherapy drug, in PDAC (28). Given more than 90% of PDAC patients retained KRAS mutations, people have made attempts to use KRAS mutations as the biomarkers for the determination of using KRAS-target antibody drugs (29). Based on these facts, we thought that the discovery of the 4 novel genes (IGSF6, TLR10, FCRL3, and IFI30) in cervical cancer might shed light on the clinical treatments of cervical cancer as well.

IGSF6 was reported to be involved in the immune regulations of atherosclerosis (30) and inflammatory bowel disease (31). TLR10 is the only member of Toll-like receptors that exerts anti-inflammatory function. TLR10 could compete for the binding of stimulatory TLRs with other TLRs and activate PI3K/Akt pathway to express IL-1Ra (32). A recent study showed that TLR10 was upregulated, thus promoting immune infiltration in breast cancer, and played an important role in tumor development (33). FCRL3 was documented to be involved in many autoimmune diseases (34). FCRL3 was able to promote IL-10 expression through the MAPK pathway in B cells (35). Meanwhile, FCRL3 contributed to the activation of B cells, while suppressing the differentiation of plasma cells (36). IFI30 has been known as a prognosis factor for many cancers, such as glioma (37, 38) and breast cancer (39). GILT, the protein encoded by IFI30, was shown to enhance the T cells mediated immune surveillance against tumor cells, implying its druggable potential in tumor therapy (40). Despite the known roles of the above four genes in regulating immune processes, the detailed mechanism of how the four genes manipulated the CD8+ T cell infiltration warrants further investigation. Apart from this, an intriguing notion is that the four genes (IGSF6, TLR10, FCRL3, and IFI30) were also upregulated in cervical cancer compared to normal tissues (**Figures 7A**, **8A**), while their high expressions predicted better prognosis (**Figures 7C–F**, **9A–C**). The profiles of the four genes highly resemble the CD8+ T cell infiltration, which agreed with their correlation tests (**Figures 7B**, **8C**). A possible explanation was that these genes were also evoked upon tumorigenesis and cooperated with CD8+ T cell infiltration, aiming at eliminating the tumor cells within the body. Under the circumstance in which the expressions of these four genes were relatively lower, the prognosis would be worse. The combination of the four genes could serve as the prediction tool for the prognosis of cervical cancer.

## Data availability statement

Publicly available datasets were analyzed in this study. This data can be found here: GEO (GSE151666) and The Cancer Genome Atlas (TCGA).

## Ethics statement

The studies involving human participants were reviewed and approved by the Ethics Committee of Anhui Normal University. The patients/participants provided their written informed consent to participate in this study.

## Author contributions

Conceptualization, XS, CW, and ML; Methodology, XS, CW, and ML; Software, XS and CW; Investigation, XS, CW, ML, SW, YZ, ZL, and GZ; Writing – original draft, XS; Writing – review and editing, XS; Visualization, XS, CW, and SW; Supervision, XS; Project administration, XS; Funding acquisition, XS. All authors contributed to the article and approved the submitted version.

## Funding

This work was supported by the National Natural Science Foundation of China (No. 31701289), Anhui Provincial Natural Science Foundation (No. 2208085MH209, 1808085QH234), Anhui Provincial Funding Scheme to Outstanding Innovative Programs by Returned Scholars (No. 2019LCX003), Anhui Provincial Key Laboratory of Molecular Enzymology and Mechanism of Major Diseases (No. fzmx202001), Educational Commission of Anhui Province of China (No. KJ2017A319, KJ2019A0498, KJ2020A0058, KJ2020A0087), Key Projects for Young and Middle-Aged People from Wannan Medical College (No. WK2021ZF08), and the Foundation for High-level Talents in Higher Education of Anhui Province of China and Funds from the Anhui Normal University (No. 2017XJJ38, start-up funds to XS).

## Acknowledgments

We thank the platforms and resources provided by Anhui Provincial Key Laboratory of Molecular Enzymology and Mechanism of Major Diseases, Anhui Provincial Engineering Research Centre for Molecular Detection and Diagnostics, Anhui Provincial Key Laboratory of the Conservation and Exploitation of Biological Resources, and Key Laboratory of Biomedicine in Gene Diseases and Health of Anhui Higher Education Institutes.

## Conflict of interest

The authors declare that the research was conducted in the absence of any commercial or financial relationships that could be construed as a potential conflict of interest.

## Publisher's note

All claims expressed in this article are solely those of the authors and do not necessarily represent those of their affiliated organizations, or those of the publisher, the editors and the reviewers. Any product that may be evaluated in this article, or claim that may be made by its manufacturer, is not guaranteed or endorsed by the publisher.
